# A Fast and Easily-Realized Concentration Sensor for Binary Gas Mixtures and Its Design Analysis

**DOI:** 10.3390/s18041257

**Published:** 2018-04-19

**Authors:** Yu Guan, Song Lu, Dan Zhang, Yang Hu, Wei Yuan

**Affiliations:** State Key Laboratory of Fire Science, University of Science and Technology of China, Hefei 230026, China; aa913958@mail.ustc.edu.cn (Y.G.); danzhang@mail.ustc.edu.cn (D.Z.); hyang611@mail.ustc.edu.cn (Y.H.); lisayuan@mail.ustc.edu.cn (W.Y.)

**Keywords:** gas concentration detection, binary gas mixtures, pressure drop, CBrF_3_, C_3_HF_7_

## Abstract

A low-cost and easily-realized sensing device used for the detection of gas mixtures at different concentrations is presented. Its sensing part includes a small critical nozzle, a laminar structure, and a differential pressure sensor. When gas flows through the laminar structure, there is a pressure drop between both ends of it, and for different components of gas, the pressure drop is different. Based on this feature, the concentration detection is achieved. Concentration tests for two types of fire extinguishing agents CBrF_3_ and C_3_HF_7_ are presented. The results show the characteristics of fast response/recovery time, high accuracy, and good repeatability. Based on the theoretical analysis, the effects of the design parameters on the sensing performance to concentration detection are discussed in detail.

## 1. Introduction

It is important to detect the gas concentration in various situations such as the detection of fire extinguishing gas agent concentration. The gas extinguishing system is widely used and exerts great influence on fire suppression due to its high efficiency and cleanliness. For the assessment of its fire-fighting effectiveness, the study of agent concentration distribution is essential. As many factors such as obstacles or convection will lead to hetaerogeneous concentration distribution, and if the agent concentration does not reach the suppression concentration in some areas, the fire can still lead to catastrophe. For the gas extinguishing system, the agent is very quickly released and dispersed, and the suppression concentration is usually at high levels such as CBrF_3_ (6%), C_3_HF_7_ (9%), and CO_2_ (50% for smoldering); pure agent is released into the air, and the concentration range can be from 0% to 100%. Therefore, to verify the effectiveness, the gas sensing technique should have the characteristics of fast response, accurate detection, and wide detection range, and due to the need of multi-point detection, the cost of each sensor should also be low. The agent is released into the air; thus, the mixture of agent with the air can be considered as a binary gas mixture. 

A number of gas concentration sensing techniques have been successfully developed, but not all of them can meet the above requirements. Metal oxide semi-conductor gas sensors are widely used due to their high material sensitivity, light weight, and robustness. George et al. reviewed the metal oxide semiconductor material for the detection of CO_2_ including ITO, SnO_2_, BaTiO_3_, and LaOCl, which showed high sensitivity; its the range is from 2000 to 10,000 ppm, while its detection range is relatively small, and it is mainly used for the detection of low gas concentrations [1,2,3,4]. The optical sensing technique has the characteristics of fast response and good selectivity [5,6]. Hu et al. introduced a NDIR sensor for the detection of extinguishing agent CBrF_3_ with a range of 0–25% and showed good accuracy, but the detection range was relatively small, and for multi-point detection the cost can be high [7]. Some sensing techniques used for binary mixtures are also developed as follows. Concentration detection based on density change is low-cost and has a wide detection range, and Sparks et al. discussed the detection of CO_2_, but unfortunately the response time is unmentioned [8,9]. Concentration detection by sensing pressure drop is studied [10,11,12]. Zhao et al. introduced an extinguishing agent detector for CO_2_ ranging from 0 to 100% with an error range within 2% FS, which was achieved by sensing pressure drop caused by viscosity variation, but it experiences trouble when the viscosity is not monotone with concentrations such as CBrF_3_ discussed in Section 4, and when the response time is not researched [13]. Yamazaki et al. described a detection system by sensing pressure drop caused by the change of density and viscosity under the same flowrate, which the response time could reach to 0.2 s, but the accuracy for detection of CO_2_ was not good, and the error was within 8.8% FS (range from 0 to 100%) [14]. Youn et al. improved Yamazaki’s device by matching different flowmeters with the errors within ±4% FS, and unlike Yamazaki’s their device was achieved by sensing pressure drop caused by density change or viscosity change, but when the density is similar and viscosity is not monotone, there will be problems [15]. For our sensing device, it was achieved by sensing the change of pressure drop caused by variation of viscosity, isentropic exponent, and molecular weight under changing flowrate, and the precision improved.

This paper presents a low-cost and easily-realized gas concentration detector for a binary gas mixture. It is used for gas extinguishing agent concentration detection, and it can also be applied to other binary gas mixtures as discussed in Section 4.2. It has the advantages of fast response/recovery time, high accuracy, and good repeatability with the whole concentration detection range. Its sensing part includes a small critical nozzle, a laminar structure, and a differential pressure sensor. The theoretical basis and calibration process for the detection are described. Special attention is given to two types of fire extinguishing agents: CBrF_3_ and C_3_HF_7_. The experiments for the mixture of CBrF_3_ with N_2_ showed good accuracy (the absolute errors are less than ±0.5% from 0 to 10%, and within ±1.5% at higher concentrations). The results also showed the response/recovery time of 0.11/0.15 s can be achieved by improving the design of the geometry structure. The experiments for C_3_HF_7_ showed good repeatability and the feasibility of the fitting model. Based on the experiment results and theoretical analysis, the effect of design parameters (i.e., geometry structure, temperature, and gas properties) on sensing performance was discussed in detail, which can be used for the sensor design and the analysis of the applicability of other gas mixtures.

## 2. Sensing Mechanism

**Figure 1:**
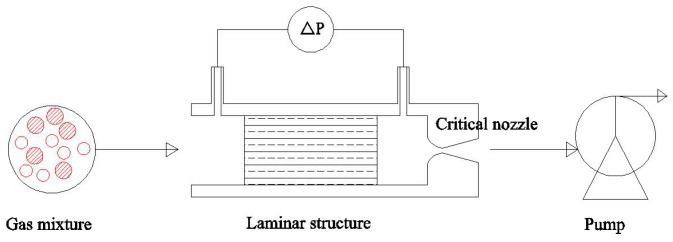
Schematic diagram of the sensing part.

In the system, gases driven by a pump passes through a laminar structure and a critical nozzle in turn (Figure 1). As gases flow through the laminar structure, the pressure drop between its ends is measured. The pressure drop of different concentrations of gas mixtures is different. Principles governing the system are based on choked flow behavior and Hagen-Poiseuille law. 

**Figure 2:**
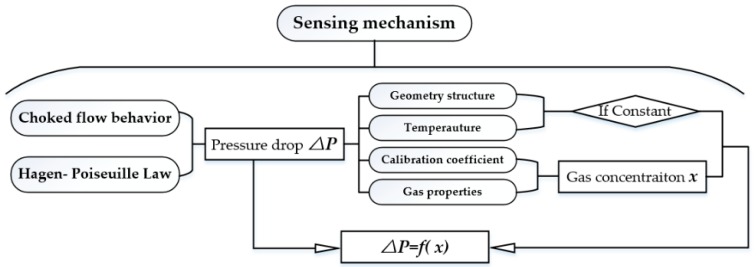
The flowchart of the analysis of sensing mechanism.

This part describes the sensing process. As shown in Figure 2, the choked flow behavior and Hagen-Poiseuille law are analyzed firstly, and based on these two principles pressure drop equation is deduced. The pressure drop is related to four parts: geometry structure, temperature, calibration coefficient, and gas properties. Then, these four parts are analyzed. For calibration coefficient, and gas properties parts, they can be expressed as a function of gas concentration, and if the geometry structure and temperature are kept as constant, the pressure drop is only related to gas concentration *x*; in other words, the pressure drop is a function of gas concentration. Therefore, according to the pressure drop, the gas concentration can be obtained.

**Figure 3:**
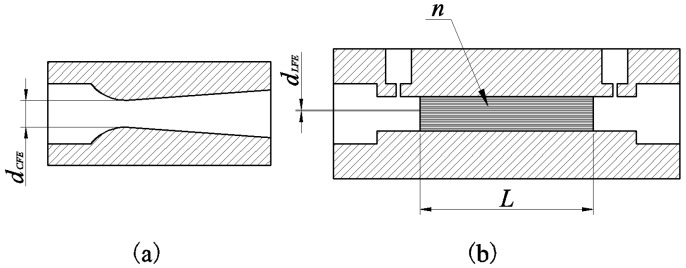
Schematic diagram of two flowmeters: (**a**) the diagram of critical nozzle, (**b**) the diagram of laminar structure).

The critical nozzle (Figure 3a) follows the choked flow behavior, a limiting condition in which the mass flow rate of fluid flowing through a restriction will never change at a given pressure and temperature as long as the pressure of the downstream of restriction is low enough [16,17,18]. The volume flow rate $Q_{V}$ through critical nozzle is given [19] as:

(1) $$Q_{V}=\frac{\pi }{4}d_{CFE}^{2}C_{d}z\sqrt{\frac{RT_{0}}{M}}\sqrt{k{\left(\frac{2}{k+1}\right)}^{\frac{k+1}{k-1}}}$$

$d_{CFE}$ and $C_{d}$ are the diameter of the throat and the discharge coefficient of critical nozzle, respectively. z is the compressibility factor of gas. R  is the universal gas constant. $T_{0}\;,\;M,$ and k are the temperature, molecular weight, and isentropic exponent of gas, respectively. It can be seen from Equation (1) that $Q_{V}$ is up to $T_{0}$ when the gas composition does not change, and it is not affected by the pressure. 

The principle of laminar structure (Figure 3b) is based on the Hagen-Poiseuille law [20]. Considering the correction to theory, viscous calibration coefficients are introduced. The equation is given [21] by:

(2) $$\Delta P=C_{\mu }\frac{8L}{\pi {\left(\frac{d_{LEF}}{2}\right)}^{4}}\frac{\mu Q_{V}}{zn}$$

in which ΔP is the pressure drop, $C_{\mu }$, *L*, $\;d_{LEF}$, and *n* are, respectively, the viscous calibration coefficient, length of pipe, diameter of pipe, and number of pipelines in laminar structure, as shown in Figure 3b. 

As the gas passes through a laminar structure and a critical nozzle in turn, so these two flowmeters have the same flow rate. Combining Equations (1) and (2), the working equation can be established as:

(3) $$\Delta P=C_{\mu }C_{d}\frac{32d_{CFE}^{2}L\sqrt{R}}{nd_{LFE}^{4}}\sqrt{T_{0}}\mu \sqrt{\frac{1}{M}k{\left(\frac{2}{k+1}\right)}^{\frac{k+1}{k-1}}}$$

Based on above analysis and Equation (3), the pressure drop is related to four parts: calibration coefficient $C_{cal}\left(C_{\mu },C_{d}\right)=C_{\mu }C_{d}$, geometry structure $\;G_{geo}\left(d_{LEF},d_{CFE},L,n\right)=\frac{32d_{CFE}^{2}L\sqrt{R}}{nd_{LFE}^{4}}$, temperature $G_{T}\left(T_{0}\right)=\sqrt{T_{0}}$, and gas properties $G_{pro}\left(\mu ,k,M\right)=\mu \sqrt{\frac{1}{M}k{\left(\frac{2}{k+1}\right)}^{\frac{k+1}{k-1}}}$. So, ΔP can be written as:

(4) $$\Delta P=G_{geo}\cdot G_{T}\cdot C_{cal}\cdot G_{pro}$$

It can be seen that $C_{cal}\cdot G_{pro}$ is relative to gas properties. The variables $k,M,\mu ,C_{\mu }$ and $C_{d}$ will vary with the concentration of gas mixtures. Hence, $C_{cal}\cdot G_{pro}$ can be expressed as the function of concentration of gas mixtures $G_{con}\left(x\right),$ and if the temperature and geometry structure is kept as constant, $G_{geo}\cdot G_{T}$ will be a constant, and the equation can be expressed in the following form:

(5) $$\Delta P_{mix}=F\left(x\right)$$

As ΔP will not be affected by the pressure behind the critical nozzle when the flow reaches the critical state [22]; the sensing parts can be arranged in a parallel manner in a system so that they will not affect each other. Therefore, the method can be used for multi-point detection. However, in this paper, the research of this method is focused on the individual, and the form of parallel structure is not discussed.

## 3. Design of Experimental Apparatus

**Figure 4:**
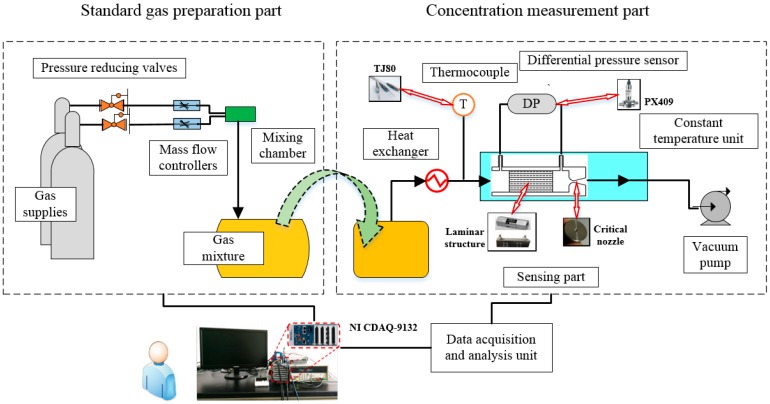
The structure diagram of experimental apparatus.

A schematic of the constructed experimental apparatus is shown in Figure 4. The system consists of a standard gas preparation part and a concentration detection part.

The standard gas preparation section is the auxiliary device is used to generate gas mixtures for the experiments. It is composed of gas supplies, pressure reducing valves, mass flow controllers, a mixing chamber, and a gas mixture storage unit (sample bags). From Figure 4, gas supplies provide the source of two pure gases, and the mass flow controllers controlled by computer set the flow rate of these two pure gases according to the needed concentration. 

The concentration detection device consists of gas sample bags, a heat exchanger, a temperature sensor, a sensing part, a constant temperature unit, a vacuum pump, and a data acquisition and analysis unit. From Figure 4, the gas mixtures in the gas sample bags flow through a heat exchanger, sensing part in sequence, driven by a vacuum pump. A differential pressure sensor is used to measure the pressure drop. The pump, on the one hand, provides the driving force to extract the gas mixtures to flow through the apparatus; on the other hand, it produces sufficient low back pressure at the downstream of critical nozzle to assure the critical state. According to discussion in Section 2, it is essential to maintain constant temperature of the gas mixtures that pass through the laminar flowmeters and critical nozzle. This is achieved by a heat exchanger and an incubator temperature control system. The gases are preheated to the operating temperature by heat exchanger and then transported to the sensing part, whose temperature constancy is maintained by incubator temperature control system, and the temperature variation is no more than 0.3 °C. All of the experimental data is collected and processed by the data acquisition and analysis unit. 

As shown in Figure 4, the critical nozzle is manufactured according to ISO 9300. Two kinds of laminar structure are used in the paper: commercial Model 50MJ10-11 (Meriam Process Technologies, Cleveland, OH, USA) and the self-manufactured one (as described in Section 4.1). The temperature sensor used is a T type thermocouple (TJ80, Omega Engineering Inc., Norwalk, CA, USA) with a range of 0–200 °C. The differential pressure sensor used in the paper (PX409, Omega Engineering Inc.) has a range of 10 in H_2_O with a output signal of 4 to 20 mA, and its accuracy is 0.08% BSL. Its response time is less than 1ms. All signals were collected by a NI CompactDAQ controller (NI CDAQ-9132, National Instrument Inc., Austin, TX, USA). 

## 4. Results and Discussion

### 4.1. Experiment Results

The experiments were conducted at a temperature of around 23 °C, and the pressure was atmosphere pressure. The temperature of the system was kept at 70 °C. For the mixture of CBrF_3_ with N_2_, samples were prepared ranging from 0 to 100%. For the mixture of C_3_HF_7_ with air, the concentration range was from 0 to 30%. The sample frequency was 1000 Hz.

The detection procedure is straightforward, as follows:

(a) Preparation of gas mixtures. The different concentration of gas mixtures is obtained by gas mixing equipment as described in standard gas preparation section.
(b) Preheating the experimental apparatus. Heat the equipment to 70 °C and maintain relatively constant temperature.
(c) Starting vacuum pump. The gases will enter the equipment, and the test begins when the temperature remains relatively constant in heat exchanger.
(d) Data acquisition and calibration. The value of pressure drop with known gas concentration is recorded by a NI CompactDAQ controller. Based on the experimental data, the calibration curve is acquired, which can be used to confirm the concentration of unknown gas mixtures in the future.

The response R is defined as the difference value of pressure drop between gas concentration 0% and x%: $R=\Delta P_{mix}\left(0\%\right)-\Delta P_{mix}\left(x\right)$. The value of 0% concentration is served as the reference value. 

**Figure 5:**
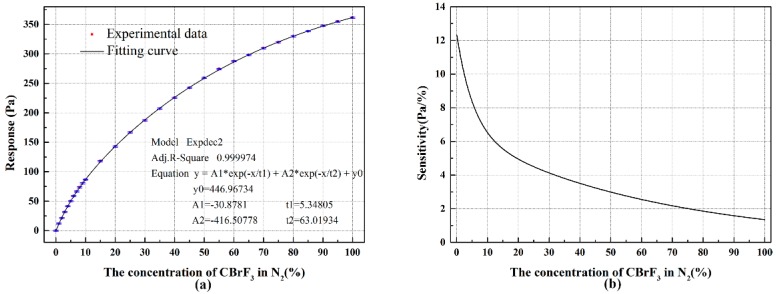
The sensing response to CBrF_3_: (**a**) the response R with the change of volume concentration of CBrF_3_ in N_2_ and (**b**) the sensitivity with change of volume concentration of CBrF_3_ in N_2_.

In the beginning, the laminar structure used in the tests is a commercial laminar flowmeter (Model 50MJ10-11), and the throat diameter of the critical nozzle is 0.88 mm. As can be seen in Figure 5a, the experimental results showed that as the volume concentration of CBrF_3_ increased, the response increased, and the monotonous trend indicates the detection is feasible. It is not convenient to use the theoretical model to fit the experimental data owing to its complexity functioning and the difficulty of acquiring precise gas properties data (the theoretical model can provide accurate predictions and instructions for sensor design as discussed in Section 4.2); a more preferable method involves using the fitting equation, and the model y = A1×exp(−x/t1) + A2×exp(−x/t2) + y0 was used, which fitted the experimental data very well, and we found it was also appropriate for other cases, as shown below. The sensitivity is defined as S=|dR/dx|. From Figure 5b, the sensitivity decreased with the increasing concentration. It indicated that the low concentration range would be more precise than high concentration range. 

**Figure 6:**
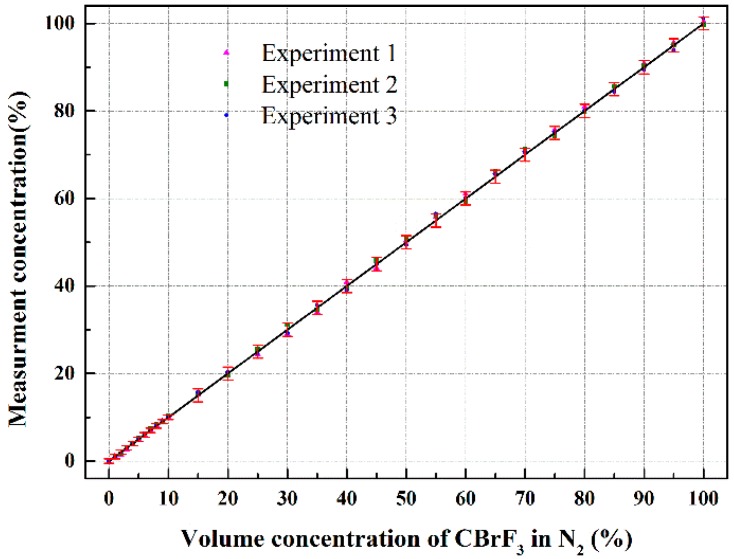
Errors in the detection of the volume concentration of CBrF_3_ in N_2_.

Three experiments were conducted. Compared with calibration curve, the absolute error with variation of the volume concentration of CBrF_3_ was shown in Figure 6. It can be found that the absolute errors are no more than ±0.5% from 0 to 10%, and at higher concentrations, the absolute errors are within ±1.5%. 

**Figure 7:**
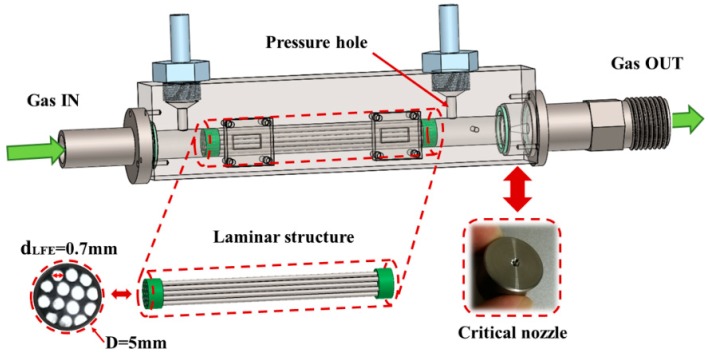
Construction of the sensing part.

Although the results by using commercial Model 50MJ10-11 showed good accuracy, the response time is not satisfied, which is around 3 s. It was caused by the design of laminar structure. To acquire quick response, a self-manufactured sensing part is designed as shown in Figure 7. The laminar structure is made by parallel capillary tubes. The inner diameter of tubes $d_{LEF}$ is around 0.7 mm, and their length is 50 mm. Fourteen pipes are used, and the diameter of the laminar structure D is 5 mm. Two sizes (0.682 mm, 0.801 mm) of the throat diameter of critical nozzle are researched. Two pressure holes are made at both ends of laminar structure with a diameter of 1 mm. To sense the pressure difference, the differential pressure sensor is connected to the pressure holes by nonmetal piezometric tubes. The sensing part is easily manufactured, and the cost is very low.

**Figure 8:**
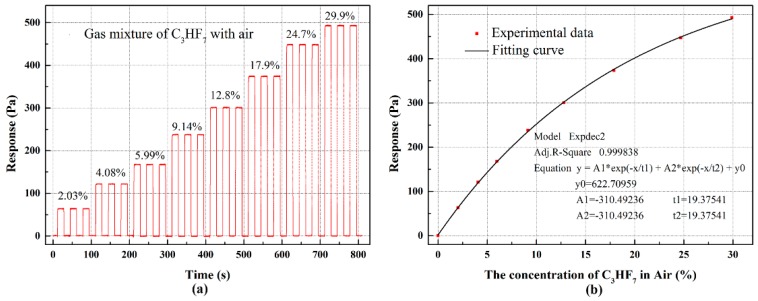
The sensing response to C_3_HF_7_: (**a**) the step response to C_3_HF_7_ in Air and (**b**) the response R with the change of volume concentration of C_3_HF_7_ in Air.

The gas mixture of C_3_HF_7_ with air ranging from 0 to 30% is researched to verify the feasibility of the self-manufactured sensing part, and the throat diameter of critical nozzle used in the experiment is 0.682 mm. The response/recovery time was measured through quickly switching gases using a solenoid valve, with a response time of less than 10 ms. From Figure 8a, it showed the evolution of the signal during the entire sequence; the detector signal could be quickly stabilized after switching the concentration. For the concentration switching between 0% and 29.9%, the time T90 (time to achieve 90% actual concentration) of response time and recovery time is 0.21 s and 0.26 s, respectively. Good repeatability was also shown. Figure 8b shows the response increased with the increasing of the concentration of C_3_HF_7_, and the fitting equation model y =A1×exp(−x/t1) + A2×exp(−x/t2) + y0  can also fit the experimental data well.

**Figure 9:**
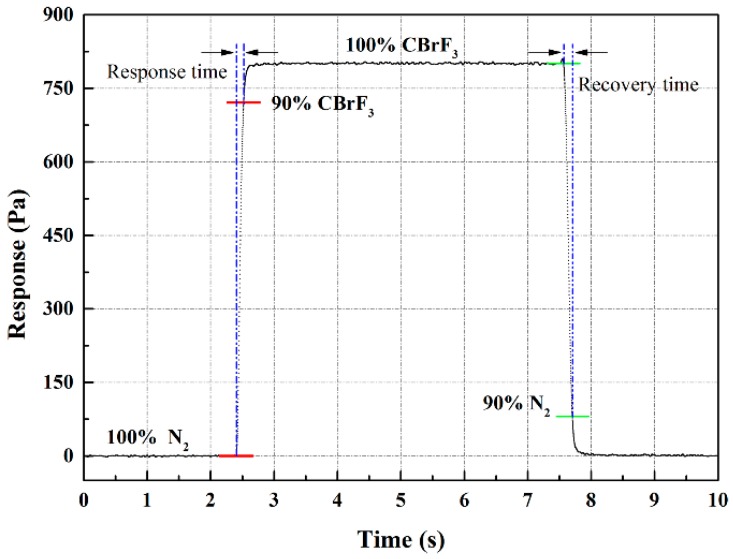
The response/recovery time of CBrF_3_ in N_2_.

To compare the response/recovery time with the commercial one, the gas switching experiment between 100% N_2_ and 100% CBrF_3_ was conducted, and the throat diameter of critical nozzle used is 0.801 mm. As shown in Figure 9, the response time is 0.12 s, and the recovery time is 0.15 s. The results show that the response/recovery time can be greatly improved by changing the structure of the sensing part. The time is correlated with geometry construction of laminar structure, including the arrangement of capillary tubes and their size (number, diameter, length); also, the shape of critical nozzle has a great influence on the response/recovery time. A more systematic approach is needed to research the effect of these parameters on time in future works.

### 4.2. The Effect of Design Parameters on Sensing Performance

Due to the shortage of precise data on the gas properties of the theoretical analysis, it is hard to fit the experimental data perfectly, which can be replaced by calibration process and can be critical for the design of the sensing equipment. According to the discussion in Section 2 and Equation (3), four parts (geometry structure, temperature, calibration coefficient, and gas properties) determine the sensing performance. For calibration coefficient $C_{cal}$, it can be considered as 1 at the design phase.

The geometry structure part is $G_{geo}\left(d_{LEF},d_{CFE},L,n\right)=\frac{32d_{CFE}^{2}L\sqrt{R}}{nd_{LFE}^{4}}$, and its effect on sensing performance is achieved by $d_{LEF},d_{CFE},L,n$. It can be seen that $G_{geo}$ will increase with the decreasing of $d_{LEF},n$ and the increasing of $d_{CFE},L$. As shown in Equation (3), $G_{geo}$ is constant once the structure is determined, and it does not change with the concentration of gas mixtures. Therefore, it is more like an amplifier; it will enlarge the distinction of different gas properties caused by gas properties part $G_{pro}$ (as discussed in the following), which will promote the sensitivity of the sensing equipment. However, that is not to say that bigger value of $G_{geo}$ is better. It should not lead to the pressure drop exceeding the range of differential pressure sensor, and for the sake of following Hagen-Poiseuille law, the flow condition in laminar structure should be laminar, which can be easily confirmed by using $Q_{V}$ to calculate Reynold number.

The temperature part is $G_{T}\left(T_{0}\right)=\sqrt{T_{0}}$. On the one hand, the temperature acts as amplifier as $G_{geo}$ promoting the sensitivity, but much smaller (in the experiment, the value of $G_{geo}$ is seven order of magnitudes, and the value of $G_{T}$ is two order of magnitudes); on the other hand, temperature also affects $G_{pro}$ via μ, k, and, especially, μ. During the experiments, as described in Section 2, the change of temperature is relatively small, so the influence of temperature on μ,k can be neglected. Hence, Equation (3) can be rewritten as:

(6) $$\Delta P\left(T,x\right)=X\left(x\right)\sqrt{T}$$

X(x) is only relevant to the concentration x and takes a derivative with respect to *T*.

(7) $$d\Delta P\left(T,x\right)=\frac{X\left(x\right)}{2\sqrt{T}}dT$$

Equation (8) is obtained by substituting Equation (6) with Equation (7).

(8) $$d\Delta P\left(T,x\right)=\frac{dT}{2T}\Delta P\left(T,x\right)$$

dT is the temperature variation. Based on Equation (8), the pressure change during the real-time detection caused by temperature fluctuation can be estimated. Additionally, it can be seen that the greater temperature is, the smaller effect of temperature variation on the pressure drop will be. Additionally, under a certain temperature, for high ΔP the change caused by temperature is bigger than low ΔP. Hence, the stability of temperature is critical to the precise detection.

Gas properties’ part is $G_{pro}\left(\mu ,k,M\right)=\mu \sqrt{\frac{1}{M}}\sqrt{k{\left(\frac{2}{k+1}\right)}^{\frac{k+1}{k-1}}}$. As mentioned above, geometry structure and temperature are like amplifier and will not change with the change of gas concentration. This part determines whether or not the sensing method is suitable for gas mixtures, and it is a co-effect of μ, k, and M, which is the function of gas concentration, so gas properties’ part can be rewritten as $G_{pro}\left(x\right)=g_{\mu }\left(\mu \left(x\right)\right)g_{M}\left(M\left(x\right)\right)g_{k}\left(k\left(x\right)\right)$. As the experimental data of μ, k, and M with change of binary gas concentration is not conveniently obtained, theoretical and empirical equation can be useful in the design phase. The isentropic exponent of gas mixtures can be calculated as followed [23], and Suffixes *i*, *j* indicate gas *i*, *j*.

(9) $$k\left(x\right)=\frac{\sum_{i=1}^{2}x_{i}C_{pi}}{\sum_{i=1}^{2}x_{i}C_{pi}-R}$$

$C_{p}$ is the isobaric heat capacity. The molecular weight of binary gas mixtures is given by

(10) $$M\left(x\right)=\overset{2}{\underset{i=1}{\sum }}x_{i}M_{i}$$

The viscosity of binary gas mixtures is obtained by using the approximate Wilke’s expression [24]:

(11) $$\mu \left(x\right)=\overset{2}{\underset{i=1}{\sum }}\frac{\mu_{i}}{1+\frac{1}{x_{i}}\sum_{\begin{array}{c} j=1\\ j\neq i \end{array}}^{2}\varphi_{ij}x_{j}},\;\;\varphi_{ij}=\frac{{\left[1+{\left(\frac{\mu_{i}}{\mu_{j}}\right)}^{0.5}{\left(\frac{M_{j}}{M_{i}}\right)}^{0.25}\right]}^{2}}{2\sqrt{2}{\left(1+\frac{M_{i}}{M_{j}}\right)}^{0.5}}$$

**Figure 10:**
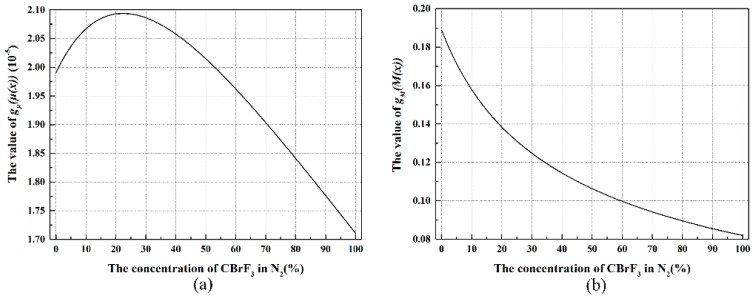

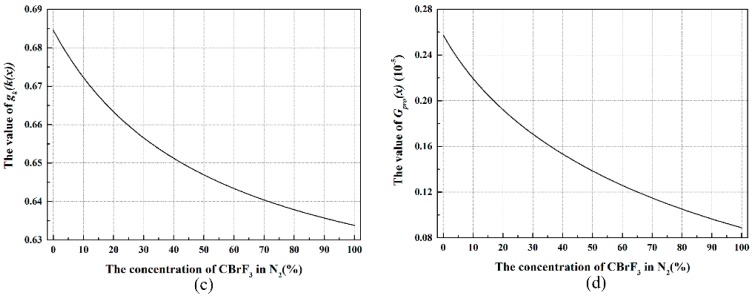
The effect of gas properties on sensing performance to CBrF_3_ in N_2_ ((**a**) the value of $\;g_{\mu }\left(\mu \left(x\right)\right)$ with change of CBrF_3_ concentration, (**b**) the value of $\;g_{M}\left(M\left(x\right)\right)$ with change of CBrF_3_ concentration, (**c**) the value of $\;g_{k}\left(k\left(x\right)\right)$ with change of CBrF_3_ concentration, (**d**) the value of $\;G_{pro}\left(x\right)$ with change of CBrF_3_ concentration).

The analysis of $G_{pro}\left(x\right)$ of CBrF_3_ in N_2_ is shown in Figure 10, and this analytical method is suitable for other binary gas mixtures. As shown in Figure 10a, the viscosity influence term $g_{\mu }\left(\mu \left(x\right)\right)$ does not monotonically decrease with concentration, but it has a peak value at around 23%. Thus, it will be not workable to measure the concentration only on the basis of the change of viscosity [13], because two concentration values may be found at the same value, and the concentration changes slowly around the peak, which results in bad resolution. It increases from 1.99 × 10^−5^ to its peak 2.09 × 10^−5^ and then decreases to 1.71 × 10^−5^. The reduction is up to 18% ((value at 0% − value at 100%)/value at 0%; the same analysis method is used for $g_{M}$ and $g_{k}$). From Figure 10b, the molecular weight influence term $g_{M}\left(M\left(x\right)\right)$ is gradually decreased by about 57%. It has a great influence on the pressure drop and greatly increases the distinction between the gas concentrations and the viscosity influence term. For the isentropic exponent influent term $g_{k}\left(k\left(x\right)\right)$, as can be seen in Figure 10c, it becomes smaller with the increase of the volume concentration of CBrF_3_ and decreases by 7%. Compared with above-mentioned two terms, its change is the smallest, but it still increases the distinction between the gas concentrations. Figure 10d shows the co-effect of these three terms. $G_{pro}\left(x\right)$ gradually decreases with the concentration of CBrF_3_. Its reduction is up to 65%, which is similar to the change of pressure drop (61%) as shown in Figure 5a. Gas properties part $G_{pro}\left(\mu ,k,M\right)$ shows the change of different gas concentration, and, based on these parameters, the tendency of gas concentration versus pressure drop can be estimated; additionally, the geometry structure and temperature is constant, and the sensitivity can be estimated as following:

$$S=\frac{d\left(\Delta P\left(T,0\%\right)-\Delta P\left(T,x\right)\right)}{dx}=\left(G_{geo}\cdot \sqrt{T_{0}}\right)\cdot \frac{d(G_{pro}\left(0\right)-G_{pro}\left(x\right))}{dx}$$

It can give good advice on whether or not the method is suitable for the other binary gas mixtures.

## 5. Conclusions

In this study, a fast and easily-realized concentration sensing device for a binary gas mixture was presented. The principles and calibration process of the system were described; two types of fire extinguishing agents, CBrF_3_ and C_3_HF_7_, are researched in the tests. Additionally, experiments for the gas mixtures CBrF_3_ with N_2_ ranging from 0 to 100% at 70 °C showed that the absolute errors are less than ±0.5% from 0 to 10%, and within ±1.5% at higher concentrations. The results also showed the response/recovery time can be achieved to 0.11/0.15 s, and time can be greatly reduced by improving the geometry structure. The experiments for C_3_HF_7_ with air showed the good repeatability and feasibility of the fitting model. According to the experimental data and theoretical analysis, the effect of the design parameters (i.e., geometry structure, temperature, and gas properties on sensing performance) were discussed in detail and can be used for the sensor design and analysis of the applicability of other gas mixtures.
